# The short-term effect of BMI, alcohol use, and related chronic conditions on labour market outcomes: A time-lag panel analysis utilizing European SHARE dataset

**DOI:** 10.1371/journal.pone.0211940

**Published:** 2019-03-11

**Authors:** Andrea B. Feigl, Yevgeniy Goryakin, Marion Devaux, Aliénor Lerouge, Sabine Vuik, Michele Cecchini

**Affiliations:** 1 Harvard T.H. Chan School of Public Health, Department of Global Health and Population, Boston, MA, United States of America; 2 Organization of Economic Cooperation and Development, Paris, France; Heidelberg University, GERMANY

## Abstract

**Introduction:**

Non-communicable diseases (NCDs) like cancer, cardiovascular disease, and diabetes have spread at a remarkable pace in European countries over the past decades. Overweight/obesity and alcohol use are two leading risk factors contributing to both economic and epidemiological burden associated with NCDs. In OECD countries, the impact of indirect costs of obesity varies between 0.20% and 1.21% of GDP. Indirect costs of alcohol use range from 0.19% (Portugal) to 1.6% (Estonia) of GDP.

**Aim:**

To assess the longitudinal impact of alcohol use and high body-mass index (BMI) on labour market outcomes in the European region by modeling the direct effect of high BMI and alcohol use, and the effect via associated diseases.

**Methods:**

The impact of BMI, alcohol use, and associated diseases on employment likelihood, intent to retire early, days of absenteeism, and hours of work per week, were modelled via lagged Poisson and Zero-inflated Poisson regressions, adjusting for missingness via inverse probability weighting, as appropriate, using European SHARE data.

**Results:**

Controlling for other chronic conditions, being overweight increases employment likelihood among men, but not among women. Obesity decreased female, but not male, employment chances. All chronic conditions linked with high BMI negatively affected employment likelihood, and increased the intention to retire early significantly. Alcohol use positively affects employment likelihood in women at all drinking levels relative to lifetime abstainers, but only in moderate (not heavy) male drinkers. There is super-additionality of impact of NCDs on absenteeism and hours worked, presenting a key economic argument to tackle NCD prevention and compression of morbidity.

**Implications:**

NCD prevention is not just important for employment and hours worked, but also for employee morale, especially given increasing retirement age in Europe and globally.

## Introduction

Non-communicable diseases (NCDs) like cancer, cardiovascular disease, and diabetes have spread at a remarkable pace in European and other OECD countries over the past decades. In addition to being major causes of ill health and premature death, NCDs jeopardize progress towards the Sustainable Development Goals and economic growth of countries at all levels of income [1]. While evidence of the health impacts of NCDs is widely available, evidence of the magnitude of economic impacts, particularly the impact on the indirect costs burden, is more limited.

Overweight/obesity and alcohol use are two of the leading risk factors contributing to the disease and economic burden of NCDs. In the most extensive and detailed review to date, it was shown that in 2015, 107.7 million children and 603.7 million adults were obese globally, and that “since 1980, the prevalence of obesity has doubled in more than 70 countries.” [2] Furthermore, overweight and obesity were directly responsible for four million deaths globally, nearly 40% of which occurred in persons who were not obese, but overweight. More than two thirds of deaths related to overweight/obesity were due to cardiovascular diseases (CVD) [2].

The harmful use of alcohol causes approximately 3.3 million global deaths annually (or 5.9% of all global deaths), and 5.1% of the global burden of disease is attributable to alcohol consumption [3, 4]. Alcohol is a causal factor in more than 200 disease and injury conditions: for example, drinking alcohol is associated with a risk of developing alcohol dependence, liver cirrhosis, and cancers. In OECD countries, alcohol consumption is about twice the world average. The related social costs are estimated in excess of 1% of GDP in high- and middle-income countries [5].

### Measuring the indirect burden of disease

The economic burden of risk factors and associated diseases can be estimated through a variety of approaches. A comprehensive review of methodologies (Figure A in S1 File) confirmed that the majority of studies on the economic burden of disease use the Cost of Illness (COI) approach [6], with origins in the 1960s [7]. Other economic impacts often modelled as part of the economic burden of diseases are the impact on economic output (as measured by GDP and GDP growth), as well as the social or welfare costs of diseases. A more detailed description of these approaches can be found in the Supporting Information S1 File. Few cross-country studies of the direct and indirect costs of obesity or alcohol exist, and methodologies and data quality vary widely across studies [8].

The aim of the herein presented body of work is to assess the impact of alcohol use and high BMI (separately) on labour market outcomes in SHARE (Survey of Health, Ageing and Retirement in Europe) countries.

To contextualize this analysis, a semi-structured review on the existing estimates of the indirect burden related to alcohol use and high BMI was conducted, including also a review on the best practice methodologies to estimate this burden (See Supporting Information S1 File). Overall, the impact of indirect costs associated with obesity varied between 0.20% (Germany 2015) and 1.21% of GDP (Germany 2016). Total indirect costs related to alcohol use ranged from 0.19% (Portugal) to 1.6% (Estonia) of GDP in the year the costs were incurred.

For the remainder of this article, the impact of obesity- and alcohol-mediated conditions in the labour market is defined as the sum of the direct impact of these conditions on labour market outcomes, as well as the impact via diseases that are causally related to these two risk factors and contribute a large share of the attributable disease burden (See Supporting Information S1 File for range of conditions). The term impact refers to the short-term (~ two years) lagged impact of the modelled exposure on the outcomes.

## Methods

A longitudinal model utilizing SHARE Release 6.0 [9–13] was used to assess the impact of lagged BMI, alcohol use, and diseases directly attributable to these risk factors on the likelihood of employment, days of absenteeism, desire to retire early, and hours of work per week. The impact of high BMI and alcohol use were modelled separately.

The impact of alcohol use and high BMI on labour market outcomes were modelled via a) the direct impact of these risk factors on labour market outcomes, controlling for the chosen NCD, as well as b) the impact of these risks via chronic conditions that are attributable to these risk factors in the EU+3 (EU member states, plus Norway, Iceland, and Switzerland) pending data availability in SHARE (Table B in S1 File). Obesity has been shown to lead to ischaemic heart disease (IHD), diabetes mellitus (DM), cerebrovascular diseases (CVDs), chronic kidney disease (CKD), low back and neck pain, hypertensive heart disease (HHD), colon and rectum cancer, osteoarthritis, oesophageal and liver cancer, and pancreatic and kidney cancers [14]–conditions that together constitute 95% of the obesity-attributable disease burden in the EU+3 countries (Table A in S1 File). Alcohol is a causal factor for cirrhosis, alcohol use disorder (AUD), road and unintentional injuries, self-harm, liver and breast cancer, CVDs, nasopharynx and oesophageal cancer, as well as IHD and DM, together constituting 95% of the alcohol-attributable burden in the EU+3 region (Table A in S1 File). Of note is the fact that whereas harmful alcohol use is positively correlated with an increase in DM and IHD, moderate alcohol consumption was found to lower the disease burden of these two conditions in some studies [15–18].

The diseases representative of 95% of the disease burden in the EU region were mapped as best possible unto existing disease categories in SHARE (Table A in S1 File); if data for these conditions were not available in SHARE, the impacts of ‘at least 1’ and ‘at least 2’ health conditions on labour market outcomes were estimated. Overall, this list of conditions, and the respective BMI and alcohol consumption categories, constituted the exposure variables in the model (Table A & Table B in S1 File). BMI was classified into underweight, normal weight, overweight, and obese, based on the standard WHO definition [19] (Table A in S1 File). Due to the small sample size in the underweight category, and that the average BMI in the underweight category was close to 18, the underweight and normal weight category were merged for all models.

Alcohol use was classified into lifetime abstainers, former drinkers, current low/moderate drinkers (male: >0 to ≤40g alcohol per day; female: >0 to ≤20 alcohol per day), and current heavy drinkers (male: >40g alcohol per day; female: >20 alcohol per day). While this standard classification was applied to all countries, the definition of what constitutes one drink differs across countries. Nevertheless, the SHARE survey defined one standard drink as containing 12g of alcohol, and informed respondents how this classification related to country specific drinking sizes, thus allowing for the construction of a variable representing the average grams of alcohol that were consumed by day, subsequently used to defined current drinking status (See Table B in S1 File).

### Data sources

SHARE is a multidisciplinary and cross-national panel database of micro-data on health, socio-economic status and social and family networks of more than 120,000 individuals aged 50 or older (more than 297,000 interviews). SHARE covers 21 European countries across Waves 1–6 (See Supporting Information S1 File for countries represented herein). Countries with at least two data points between Waves 1 to 6 (except Wave 3, which is a retrospective dataset) constituted the main dataset for this analysis (Table C & Table D in S1 File), covering years 2004–06, 2006/07, 2010/11/12, 2013, and 2015, respectively. Panel data between two consecutive waves constituted the data for the analysis.

Hungary and Croatia only participated in one wave, thus were excluded from the analysis; Greece did not participate in Wave 4 and 5, and is thus only included in a subset of analyses; The Netherlands did not participate in Wave 6, and is thus only represented in a subset of analyses, including the alcohol-specific analyses; Poland did not participate in Wave 5, and thus is not included in the final results.

The age restriction for inclusion in the longitudinal analysis was 50–63 years. This reflects the fact that in the subsequent wave, exiting employment was most likely due to age-related retirement, rather than due to health status. Table 1 shows several characteristics of the study population, with sample size ranging from 27,395 to 10,490, employment rates between 55.1 to 65.8 percent, and the majority of participants female. The average population is overweight, and the percent of respondents with at least one NCD rises with the year of the survey, as does the percentage of respondents with at last two NCDs (data not weighted).

**Table 1:** Study population characteristics.

	n eligible population	Mean Age	% employed	% female	% ≥1 NCD	% ≥2 NCDs	% obese	% heavy drinkers	% tertiary education	% physically active	% homeowners	% in partnership (married, etc.)
Absenteeism Analysis:												
Wave 1 (baseline)	14,905	56.4	55.1	53.9	37.6	9.1	19.3	NA	23.5	68.9	75.8	NA
Wave 2 (follow-up)	10,490	58.9	54.6	54.9	48.4	15.3	21.3	9.2	24.4	68.0	78.5	81.9
All alcohol-related analyses:												
Wave 4 (baseline)	23,628	57.0	59.1	56.3	45.2	13.5	24.5	9.2	24.2	66.2	76.8	82.6
Wave 5 (follow-up)	17,107	59.1	58.1	56.9	53.4	19.3	24.4	8.8	24.9	67.0	78.1	79.5
Employment Likelihood & Hours worked analysis:												
Wave 5 (Baseline)	27,395	57.3	65.8	56.2	46.8	14.8	22.6	NA	27.2	68.4	77.5	79.9
Wave 6 (follw-up)	19,688	59.4	61.9	57.0	54.2	19.9	24.0	NA	27.3	66.7	78.1	80.9

A two-time-period panel analysis (rather than a multi-time point panel analysis) was performed, driven by the specific age range of the study population, as well as the >50% attrition over three or more survey waves. While missingness patterns between waves 1 and 2, and waves 5 and 6 were found to be at random for all outcomes variables in question, loss-to-follow-up between waves 4 and 5 was non-random in the overall analysis. Thus, wherever possible, waves 5 and 6 were used for the analysis, except for the alcohol module and Dutch data, where waves 4 and 5 were used, adjusted for attrition. For the impact of alcohol use on labour market outcomes, analysis was performed on wave 4 and 5 data, since wave 5 and 6 had different alcohol variables, incompatible for comparison with previous waves (Table C in S1 File).

### Variables

The main outcome variables were employment status (binary), days of work missed in the last 365 days (continuous), average hours worked per week (if working), and desire to retire early (yes/no) if working.

Exposure variables were the presence/ absence of select chronic conditions and risk factors in the previous wave, including: cancer (yes/no), diabetes (yes/no), weight category (normal/overweight/obese), hypertension (yes/no), stroke (yes/no), heart disease (yes/no), lung disease and COPD (yes/no), alcohol use (former/never/moderate/high drinker), smoking (yes/no) and level of physical activity (none/low/moderate/high categories). Lagging risk factor and disease variables served to address the temporality issue between exposure and outcomes. For alcohol use, contemporaneous drinking status and its impact on absenteeism and hours worked was modelled, also (Table C in S1 File). All variables were self-reported.

Control variables (potential contemporaneous confounders) were not lagged, and included smoking status, education level (no secondary/secondary/tertiary), age (in five-year/10-yr categories), marital status (married or in partnership/ single; the category ‘single’ included both those who were previously married, widowed, or single), gender, and number of social or religious activities per year (for the alcohol module). Current smoking levels and physical activity levels also served as control variables in select models. In general, risk factors and disease variables that acted as potential confounders were not lagged (i.e. heart disease when impact of alcohol use was investigated). The model also included country level fixed effects.

### Analytical approaches

The main model to estimate employment likelihood and intention to retire early was a modified Poisson regression (specifying the Incidence Rate Ratio (IRR) and robust standard error options), estimating the lagged health outcomes of wave *t-1* on labour market variables in wave *t* (Table D in S1 File). The modified Poisson regression has the advantage of approximating relative risks, a more interpretable measure than the odds ratio of a logistic regression; by specifying robust standard errors, any potential variance overestimation is addressed [20, 21] (See Supporting Information S1 File for further model choice justification).

The absenteeism and hours worked models were estimated using a zero-inflated Poisson (ZIP) regression [22], due to right-skewed distribution of days missed among the employed, and both those ill / not ill with a chronic condition in question had a non-zero probability of zero absenteeism. Given over dispersion and a continuous outcome variable, a ZIP regression is recommended [23]. The Vuong test [24] confirmed the model choice (see Table D in S1 File for exact specification).

### Sample weights and missingness patterns

#### Survey weights

Based on the Akaike information criterion (AIC) tests [25], and robustness checks of effect size estimates [26], it was concluded that a specification without calibrated survey weights resulted in more robust and concise effect sizes as well as improved the goodness of fit of all models (results available upon request).

#### Exploration of missingness patterns

Missingness patterns were explored for each consecutive wave.

Specifically, for all wave combinations and outcomes, the Nijman & Verbeek test was performed to assess whether missingness was at random [27]. Such specifications have been applied in SHARE analysis in previous studies, such as [28] or [29]. Based on both comparative baseline and stepwise regression analysis [27], attrition between waves 5 to 6 was judged to be random for the outcome variables in question. Thus, for the analysis of employment status and absenteeism for these two waves, no further adjustment was required, allowing for a wider selection of models. In contrast, attrition between waves 4 and 5 was non-random with respect to employment status, health status, and absenteeism for the alcohol module. Thus for the alcohol module and the Dutch employment module, attrition was adjusted for via stabilized IPW (Inverse Probability Weights)–see Supporting Information S1 File for further details and specification (Table D in S1 File). All analyses were conducted in STATA 15.

## Results

### Risk factors and employment likelihood

**Fig 1:**
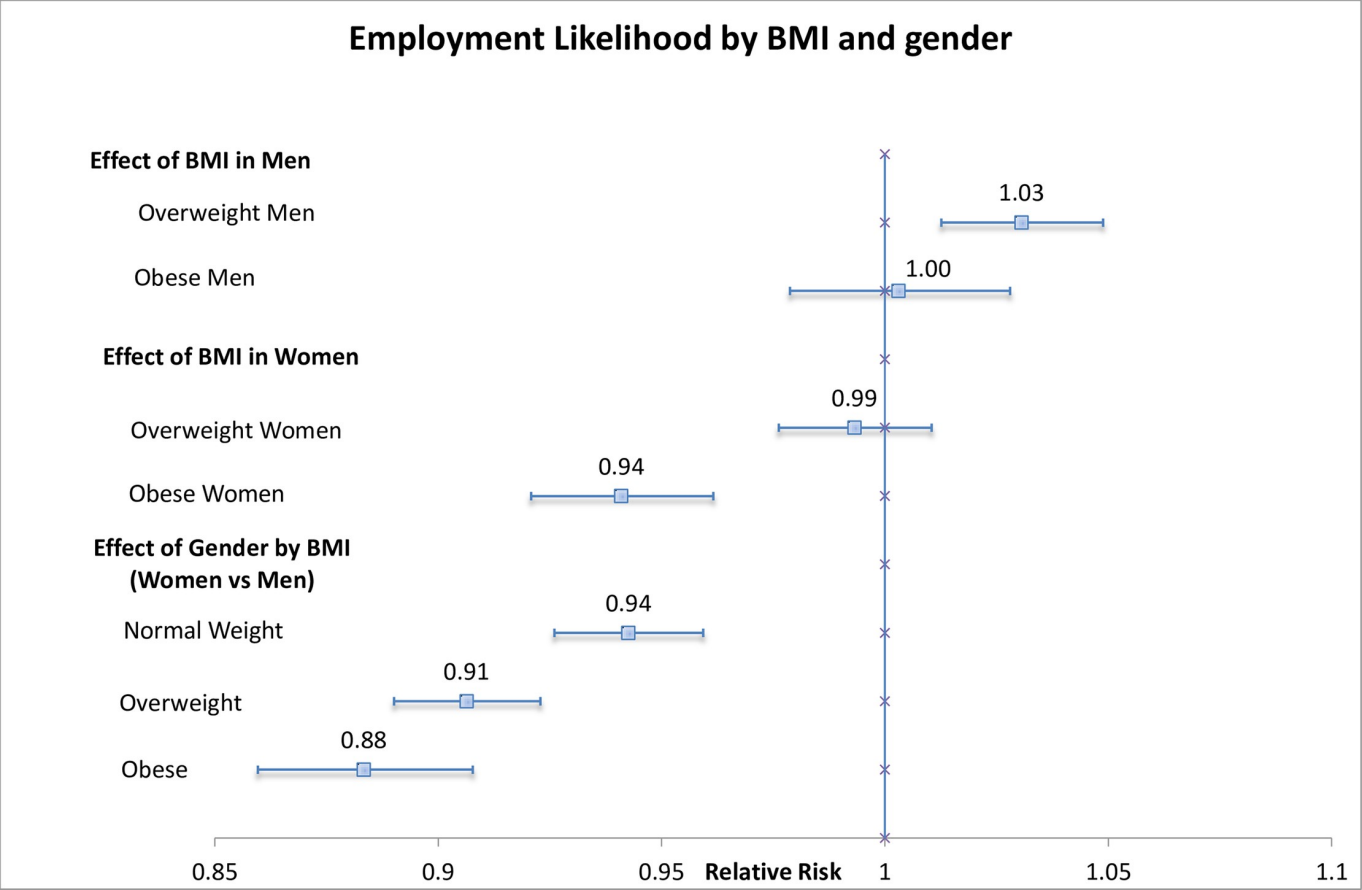
Relative Risk of being employed by BMI category. *Note*: The results are shown as relative risks (RRs), approximated from the Incidence Rate Ratios in the corresponding Poisson models. The model was adjusted for marital status, age, education, country level fixed effects, level of physical activity, smoking, drinking, and other chronic diseases. All relative risks can be combined on an additive scale, and refer to the likelihood of being employed when in the exposure category, compared to the reference category. A relative risk >1 means a greater likelihood of being employed, and a RR <1 means a lower likelihood of employment. p-value for effect modification by gender: p<0.0001 *Source*: OECD analysis of Harmonized SHARE + SHARE employment module, Release 6.0. The forest plot was created based on a template by [30].

Overall, and after adjusting for the presence of diseases, men who were overweight in the previous SHARE wave had a significantly higher likelihood of subsequent employment than previously normal weight men. A BMI ≥30 had no significant impact on employment likelihood in men, thus obese and normal weight men experienced similar employment likelihood. Being overweight versus normal weight did not impact employment likelihood among women, but obese women were 5.8% less likely to be employed than their normal weight counterparts (RR: 0.942, 95% CI: 0.92 to 0.96) (Fig 1). There was significant effect modification by gender (p<0.0001), a result especially noticeable when comparing employment likelihood among obese men and women, where obese women were only 88% as likely to be employed as obese men (RR: 0.88; 95%CI: 0.86 to 0.91). No effect modification by age was observed for this relationship. Notably, while the relationship between BMI and employment likelihood holds in country-specific results, due to lower sample size, these relationships are not consistently statistically significant in all countries.

While country specific employment probabilities differed (results from the fixed effects part), employment probabilities were consistently highest in overweight men, and lowest in obese women. Overall employment probabilities are consistently lower among women, in all modelled countries (Figure B in S1 File). In Denmark, Germany, France, Sweden, and Switzerland, obesity significantly reduced employment probability, in to both normal weight women and men. Austria, followed by Italy, had the lowest employment likelihood among obese women. Swiss and Swedish women had the highest employment probability compared to obese women in all other countries if they suffered from obesity in the previous wave.

The effect modification of education on the relationship between high BMI and employment was also explored (results not shown). The interaction was significant for obesity, but not for those overweight: at tertiary or higher education levels, those obese had a 7.5% higher (95%CI: 2.2% - 12.6%) likelihood of being employed than those obese but with lower than tertiary education.

**Fig 2:**
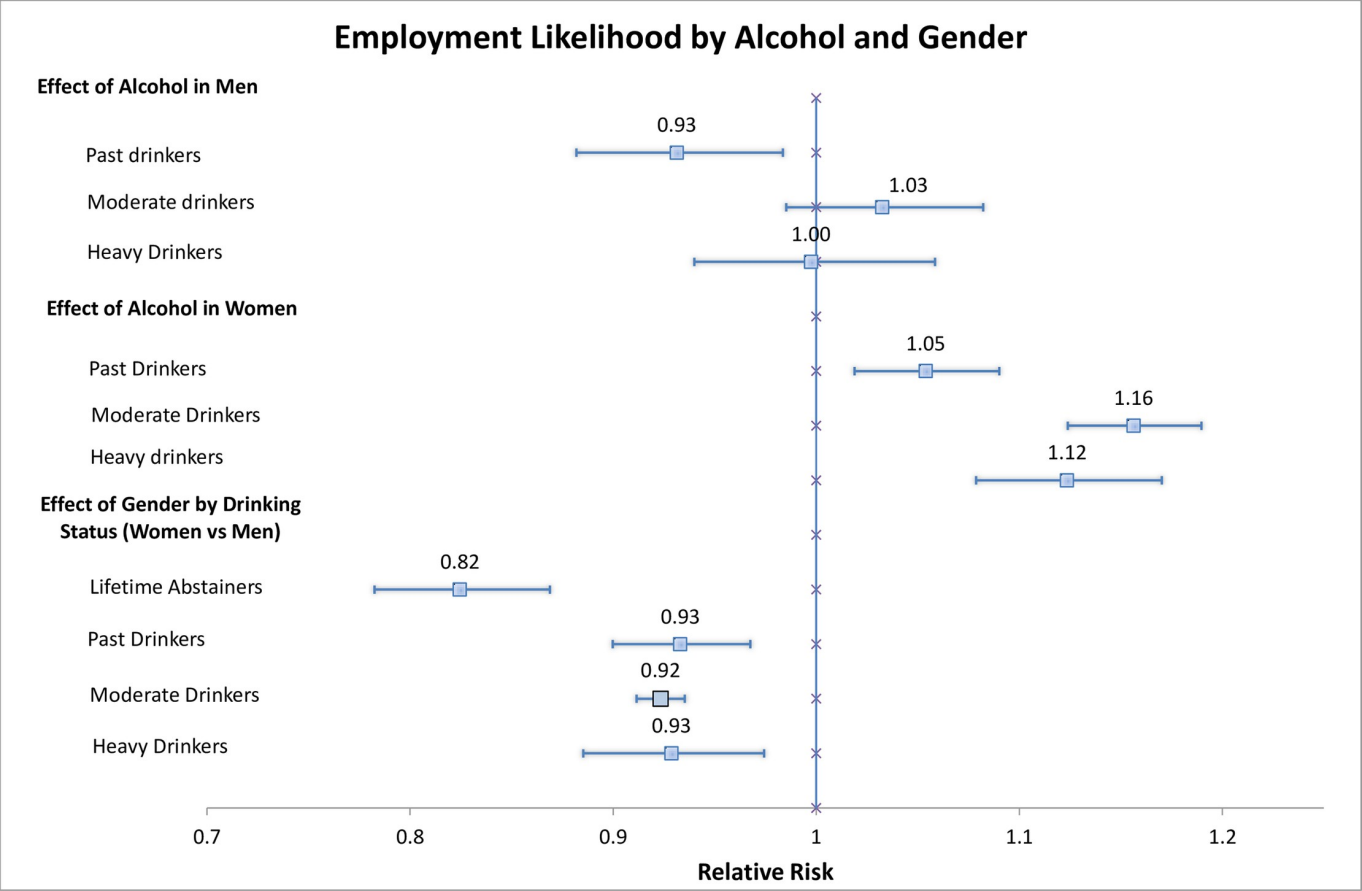
Employment Likelihood due to past drinking status. *Note*: Models adjusted for marital status, education, smoking, age, gender, country level fixed effects, and number of social events attended. p-value for gender effect modification: p<0.0001. The forest plot was created based on a template by [30].

Overall, past (previous wave’s) drinking behaviour had an overall moderate direct effect on employment likelihood two years later. Past wave drinking behaviour only impacted male past drinkers significantly; moderate and heavy drinking did not have a significant effect among men, when compared to lifetime abstainers. Past male drinkers had a 7% (95% CI: 1.7% to 12%) lower chance of employment than male lifetime abstainers (Fig 2).

Interestingly, any level of drinking (past, moderate, heavy) impacted employment likelihood positively among females, when compared to female lifetime abstainers. This positive effect on employment was strongest among moderate drinkers, with a relative risk of employment of 1.16 (95% CI: 1.12 to 1.19). There was significant effect modification by gender for the relationship between past drinking and employment (p<0.0001). Additional adjustment for engaging in religious activities (after adjusting for participation in social events) did not impact the results.

The impact of the previous survey period’s alcohol use on working likelihood in the current period was further investigated by restricting the sample to only those working in the previous wave (wave 4) and age-eligible for employment in the current wave (wave 5). This was done in order to test whether the observed impact of alcohol use on employment likelihood reflects a potential selection effect, rather than the impact of alcohol use itself. In the restricted model, none of the observed effects of alcohol use on employment remained significant, both in men and women (results not shown). Therefore, while alcohol use impacts transition into employment, it does not impact exit from employment.

### Chronic conditions and employment likelihood

Suffering from Hypertension (RR = 0.94, 95% CI: 0.919 to 0.964), Diabetes (RR = .86, 95% CI: 0.818 to 0.902), Cancer (RR = .89, 95% CI: 0.841 to 0.934), Lung Disease (RR = 0.84, 95% CI: 0.794 to 0.891), Heart Disease (RR = .84, 95% CI: 0.795 to 0.885), or Stroke (RR = .77, 95% CI: 0.703 to 0.849) in the past all impacted employment likelihood negatively (Table 2).

**Table 2:** Impact of chronic conditions on modelled outcomes.

	Employment	Additional Days Missed/year	Additional Hours Missed/week	Intention to Retire Early
Hypertension	0.94 (0.92 to 0.96)****	-0.4 (-2.21 to 1.36)	-0.48 (-0.99 to 0.03)	1.06 (1.014 to 1.11)*
Diabetes	0.85 (0.81 to 0.90)****	7.4 (2.11 to 12.8)**	0.28 (-0.69 to 1.25)	1.09 (1.00 to 1.18)*
Cancer	0.88 (0.84 to 0.93)****	6.8 (0.01 to 13.6)**	-1.39 (-2.56 to -0.214)*	1.09 (0.98 to 1.20)
Lung Disease	0.84 (0.79 to 0.88)****	3.1 (-2.2 to 8.4)	-.95 (-2.01 to 0.09)	1.11 (1.00 to 1.22)*
Heart Disease	0.85 (0.80 to 0.89)****	5.1 (0.43 to 9.86)*	-1.19 (-2.19 to -0.18)*	1.10 (1.00 to 1.20)*
Stroke	0.78 (0.71 to 0.85)****	7.1 (-7.7 to 22.1)	-2.56 (-4.48 to -0.64)**	1.16 (1.00 to 1.34)*
≥1 NCD	0.84 (.82 to .86) ****	2.72 (0.92 to 4.51)**	-0.52 (-.86 to -.18)**	1.15 (1.11 to 1.20)**
≥2 NCDs	0.70 (.67 to 0.74)****	6.98 (3.02 to 11.0)**	-1.72 (-2.31 to -1.13)***	1.18 (1.11 to 1.25)**

*Note*: The results are shown as relative risks, approximated from the Incidence Rate Ratios in the corresponding Poisson models. The model was adjusted for gender, marital status, age, education, country level fixed effects, and BMI. All relative risks can be combined on an additive scale, and refer to the likelihood of being employed when in the exposure category, compared to the reference category. No effect modification by gender was observed. A relative risk >1 means a greater likelihood of being employed, and a RR <1 means a lower likelihood of employment. For example, a person with hypertension is 0.94 times as likely to be employed as a person without hypertensions, all else being adjusted for. Note the following levels of significance

*p<0.05

**p<0.01

***p<0.001

****p<0.0001

Women with COPD had significantly lower employment likelihood than their female counterparts without the disease, in all modelled countries. However, the overall impact of the disease was stronger in men than in women (Fig C in S1 File; p for effect modification: <0.05). Female diabetics were more strongly impacted by the disease than their male counterparts, an effect that was significant in the overall model, and had the lowest employment probability, a result that is significant compared to diabetic men in Estonia, Germany, Sweden, Switzerland, Denmark, Belgium, and Czech Republic in the country-specific analysis (Fig G in S1 File).

### Days and hours of work missed due to a health condition

Weight status and alcohol consumption did not affect absenteeism in a statistically significant manner, nor did level of alcohol use significantly impact hours worked (results not shown). Three conditions that have a statistically significant effect on the marginal days at work missed among the working population, however, are diabetes (7.4 days; 95% CI: 2.11 to 12.8), cancer (6.8 days; 95% CI: 0.01 to 13.6), and heart disease (5.1 days; 95% CI: 0.43 to 9.86) (Table 2; Fig I through Fig K in S1 File). Country-specific impact of COPD, hypertension, cancer, IHD, and diabetes on days of absenteeism among its work force vary and are displayed in the Supporting Information, Fig I through Fig K in S1 File. Both for DM (15.4 days, 95% CI: 8.6 to 24.4) and cancer (14.2; 95%CI: 7.9 to 20.5), Spanish workers miss most days of work compared to workers in other countries, respectively; Fig I through Fig K in S1 File). Germany, Belgium, Denmark, and Spain have the highest number of days missed due to both of these conditions. Absenteeism is not affected by current nor previous alcohol use.

Investigating the effect of at least one or two diseases on the days of work missed, ≥1 NCD or more resulted in 2.72 days missed (95% CI: 0.92 to 4.5), and ≥2 NCDs led to almost seven days of missed work (6.98 days, 95% CI: 3.02 to 11.0) (Table 2).

Additional analyses on the difference of hours of work per week revealed that, in the past presence of at least one or two chronic conditions led to -0.52 hours (95% CI: -0.86 to -0.18) of work per week, and the presence of at least two NCDs in the past wave resulted in -1.72 hours of work per week (95% CI: -2.31 to -1.13 hours) (see Table 2). This reveals a super-additionality in the presence of an additional NCD on the impact of hours worked per week, as well as on absenteeism.

### Chronic conditions and intent to retire early

Intention to retire early was not directly impacted by heavy alcohol use in the past two years. Only obese women (vs. normal weight women) showed a significantly higher likelihood of intending to retire earlier; overweight women and overweight/obese men did not differ in their intentions to retire early from their normal weight peers (Fig H in S1 File).

However, past presence of chronic conditions impacted intention to retire early significantly. All modelled chronic conditions were positively associated with a wish for early retirement among the working population. The effect was strongest in the presence of a past stroke (RR: 1.16, 95% CI: 1.00 to 1.34), and comparatively lower in past presence of cancer (RR: 1.10, 95% CI: 1.01 to 1.21). The presence of at least one chronic disease led to a 15% higher chance of expressing interest in early retirement (95% CI: 11% to 20%); this effect was higher in the presence of at least two chronic diseases (18% increased likelihood, 95% CI: 11% to 25%) (See Table 2).

## Discussion

Overall, this study shows that key chronic conditions caused by harmful alcohol consumption and high BMI, including hypertension, IHD, stroke, cancer, DM, and COPD significantly impact labour market outcomes. On the other hand, once that the baseline presence of chronic conditions is taken into account, high BMI and alcohol consumption generally lose any statistically significant negative effect on labour market outcomes, suggesting that the diseases caused by these risk factors, rather than the risk factors themselves play the strongest harmful role. A notable exception is high BMI in women which reduces the probability of being employed, even after accounting for the presence of NCDs at baseline.

The results, while aiming to take into account the temporality between exposure and outcomes, reflect the short-term (2–3 year) consequences of the modelled conditions and risk factors. For labour market policies and planning, both short-term and long-term consequences of chronic disease risk factors and diseases are important. Further, the onset of a chronic condition might impact labour market outcomes more strongly over a longer period of time. Notwithstanding, the herein presented analyses highlights windows of opportunity and evidence to support that lowering chronic disease risk factors and managing chronic diseases might positively impact the labour market in a period of two or three years.

For the association between BMI and employment, obese women were more negatively impacted than obese men, as well as compared to their normal weight female peers. This and similar phenomena have been reported previously: Caliendo and Lee reported that obese women [compared to normal weight women] experience more negative labour market outcomes despite making greater efforts in job search and job training [31]. Overweight and obesity also increase absenteeism to a greater extent in women compared to men [32]. Other studies have also shown that a high BMI leads to direct wage penalties in women, but not in men [33]. Thus, our results, in alignment with the current literature, suggests that addressing this gap via anti-discriminatory measures might lead to lower negative impacts of obesity in the workplace for women.

Conversely, alcohol use–which often correlates with social network strength–more positively affected women’s short term chances of employment, compared to men. However, it should be noted that these results only represent the association between alcohol use and employment, and do not incorporate any potential negative effect produced by diseases caused by harmful alcohol drinking. In addition, the effects of drinking did not remain significant when the analysis was restricted to those working in the previous wave. Therefore, the observed results warrant a note of caution, that those who drink moderately may be those more engaged in society and hence, potentially, more likely to be employed. Once that these individuals join the labour force, the short-term effect of alcohol use is no longer significant. Another issue to consider while interpreting the results is that that those suffering from severe AUD are notably likely to participate in such surveys, and alcohol use in self-reported studies is most commonly underreported (See [34] for a detailed review).

When compared to existing literature, the herein presented results are similar, as the evidence on the relationship between employment outcomes and alcohol use remains inconclusive [35]. While the findings from Boden et al supported both social causation and social selection arguments [36], other studies showed that alcohol use did not impact employment [35], but that heavy drinking was associated with underemployment [37]. Some studies pointed towards an inverted U-shaped relationship between drinking and employment (moderate drinkers most likely to be employed)[38], whereas other studies failed to show such correlation [35]. Another interesting finding was that heavy drinking was positively associated with employment, but past drinking was negatively associated with employment. One potential explanation could be that past drinking might reflect social drinking while employed (i.e. during social outings), that subsequently subsided once the individual left the work force.

Past research suggests that alcohol consumption is pro-cyclical (declines with unemployment)[39], and might drop during transition from employment to unemployment[40]. Given only two successive time points in this analysis, further research, especially based on multi-year panel studies and including larger share of the population (as this study is only focused on individuals aged 50–63), is needed to more conclusively determine the relationship between alcohol use, employment, and productivity.

Of all investigated NCDs, (chronic) hypertension only had a small, borderline significant effect on employment overall. At the country level, results were only significant for Denmark (Fig G in S1 File). This is reasonable, since hypertension is relatively easily managed with lifestyle modifications and medication. Notably, this model only estimated the direct effect of hypertension, controlling for downstream CVDs, thereby causing a perhaps lower than anticipated effect. The same applies to the results for BMI on employment–while the direct effect is relatively small, the effect of BMI is mainly mediated by heart disease and other downstream chronic conditions.

While the likelihood of employment is hampered significantly if workers experienced any of the modelled chronic diseases, if they remain in the work force, there is a higher desire to retire early in the presence of all conditions, compared to the absence of these conditions (except cancer). Thus, there is not only a significant impact on labour force participation, but presumably, employee morale, as signalled with this early retirement sentiment–a finding confirming similar results by Bonsdorff et al. [41]. As pension reforms progress in countries, working more years means a workforce with higher rates of NCDs (or multiple NCDs)–as evidenced in Table 2. Investment in primary prevention, and in the prevention of co-morbidities will be essential in workforce planning of advanced economies.

One heavily relevant and little-researched question is whether the presence of two or more NCDs impact either direct or indirect costs in a manner that is greater than the sum of all individual costs–referred to as super-additionality. While such super-additionality was not present for the impact on employment likelihood and the intent to retire early, this study revealed potential super-additionality in the presence of more than two NCDs on the impact of hours worked per week and on the days of work missed per year. These findings reveal the importance of primary prevention and the development of co-morbidities. In a similar vein, the impact of multiple chronic conditions on health costs revealed super-additionality for 41 cases over 45 possible comorbidities of 10 chronic conditions in a recent study in France [42]. For example, the treatment cost for diabetes without comorbidity was estimated at 1776 € per person, whereas the diabetes-specific cost was 2634 € for people with heart disease as a comorbidity. It follows that preventing co-morbidities may be economically even more important than preventing the first chronic condition. In France, over one quarter of people suffering from a chronic condition also suffer from a co-morbidity [42]; in the Netherlands, comorbidity rose to 17.5% of the adult population in 2011 [43]; in this study, across the modelled countries in Wave 2 (Austria, Belgium, Czech Republic, Denmark, Estonia, France, Germany, Italy, Netherlands, Poland, Portugal, Spain, Sweden, Switzerland, Luxembourg, Slovenia), 14.8% of respondents suffered from the modelled co-morbidities (see Table 1).

In long-term workforce policy planning, comprehensive wellness programs, or even interventions to reduce sedentary time could be beneficial to compress (co)-morbidities, targeting potentially those at greatest risk of developing an additional NCD.

This study had several limitations. Given the small number of people that completed more than two survey waves, only a limited panel data analysis was conducted, thus lowering the claim on a truly causal relationship over multiple waves. Thus, results of this study should be interpreted as the short-term effects of alcohol consumption, high BMI and key associated NCDs on labour market outcomes, leaving scope for research on their effects on the longer term (i.e. 3 years and over). Due to data constraints, it was not possible to adjust for pension reforms and types of leave and welfare policies when modelling absenteeism. With respect to the alcohol model, the data structure and available data did not allow to fully determine the contribution of the social network effect and selection effect of the observed impact of alcohol use on increased employment. An additional limitation was that the analysis was not adjusted for self-reporting bias, which particularly affects socially sensitive variables like BMI and alcohol use. Similarly, as other datasets, SHARE may struggle to capture individuals with AUDs or other conditions caused by alcohol consumption or high BMI, therefore this analysis may underestimate their true effect on labour market outcomes. Future analyses may also include further investigation of country clusters with respect to the impact of NCDs and risk factors on the labour market, as the impact may also be reflective of, or driven by, country-specific welfare policies [44]. Similarly, other analyses may include younger population groups significantly contributing to the labour force (i.e. individuals aged between 18 and 50).

Concluding, the findings add to the growing body of literature highlighting the negative effects of NCDs and associated risk factors on the economy. Here, the results show that BMI directly, and alcohol and BMI indirectly decrease employment likelihood, increase absenteeism, decrease weekly hours worked, and increase the intent to retire early. Addressing and preventing co-morbidities will be extremely important to avert the negative impacts of super-additionality of NCDs in the workplace.

## Supporting information

S1 File Additional methods and results on measuring the indirect economic burden of disease. (DOCX)

## References

[1] Ghebreyesus TA. Acting on NCDs: counting the cost. Lancet. 2018;391(10134):1973–4. 10.1016/S0140-6736(18)30675-5 . 29627165

[2] Obesity Collaborators G. B. D. Health Effects of Overweight and Obesity in 195 Countries over 25 Years. N Engl J Med. 2017;377:13–27. 10.1056/NEJMoa1614362 . 28604169 PMC5477817

[3] World Health Organization. Global status report on alcohol and health 2014. Geneva: WHO, 2015.

[4] World Health Organization. Global status report on noncommunicable diseases 2010. Geneva: WHO, 2011.

[5] (ed) SF. Tackling Harmful Alcohol Use: Economics and Public Health Policy Sassi F, editor. Paris: OECD Publishing; 2015.

[6] World Health Organization. WHO guide to identifying the economic consequences of disease and injury. Geneva: 2009.

[7] Rice NE, Lang IA, Henley W, Melzer D. Common health predictors of early retirement: findings from the English Longitudinal Study of Ageing. Age and Ageing. 2011;40:54–61. 10.1093/ageing/afq153 21148324

[8] Withrow D, Alter DA. The economic burden of obesity worldwide: a systematic review of the direct costs of obesity. Obesity reviews: an official journal of the International Association for the Study of Obesity. 2011;12(2):131–41. 10.1111/j.1467-789X.2009.00712.x . 20122135

[9] Börsch-Supan A. Survey of Health, Ageing and Retirement in Europe (SHARE) Wave 6. Release version: 6.1.0. 2018 [cited 2018].

[10] Borsch-Supan A, Brandt M, Hunkler C, Kneip T, Korbmacher J, Malter F, et al Data Resource Profile: the Survey of Health, Ageing and Retirement in Europe (SHARE). Int J Epidemiol. 2013;42(4):992–1001. 10.1093/ije/dyt088 23778574 PMC3780997

[11] Malter F, Börsch-Supan A. SHARE Wave 4: Innovations and Methodology. Munich: MEA: Max Planck Institute for Social Law and Social Policy; 2013.

[12] Malter F, Börsch-Supan A. SHARE Wave 5: Innovations & Methodology Munich: MEA: Max Planck Institute for Social Law and Social Policy; 2015.

[13] Malter F, Börsch-Supan A. SHARE Wave 6: Panel innovations and collecting Dried Blood Spots Munich: Munich Center for the Economics of Aging (MEA); 2017.

[14] Collaborators GBDRF, Forouzanfar MH, Alexander L, Anderson HR, Bachman VF, Biryukov S, et al Global, regional, and national comparative risk assessment of 79 behavioural, environmental and occupational, and metabolic risks or clusters of risks in 188 countries, 1990–2013: a systematic analysis for the Global Burden of Disease Study 2013. Lancet. 2015;386(10010):2287–323. 10.1016/S0140-6736(15)00128-2 26364544 PMC4685753

[15] Shield KD, Parry C, Rehm J. Chronic diseases and conditions related to alcohol use. Alcohol research: current reviews. 2013;35:155–73. . 24881324 10.35946/arcr.v35.2.06 PMC3908707

[16] Shai I, Wainstein J, Harman-Boehm I, Raz I, Fraser D, Rudich A, et al Glycemic effects of moderate alcohol intake among patients with type 2 diabetes: a multicenter, randomized, clinical intervention trial. Diabetes care. 2007;30:3011–6. 10.2337/dc07-1103 . 17848609

[17] Gepner Y, Golan R, Harman-Boehm I, Henkin Y, Schwarzfuchs D, Shelef I, et al Effects of Initiating Moderate Alcohol Intake on Cardiometabolic Risk in Adults With Type 2 Diabetes. Annals of Internal Medicine. 2015;163:569 10.7326/M14-1650 26458258

[18] Global Burden of Disease Collaborative Network. Global Burden of Disease Study 2016 (GBD 2016) Incidence, Prevalence, and Years Lived with Disability 1990–2016. Lancet. 2017;390:1211–59. 10.1016/S0140-6736(17)32154-2 28919117 PMC5605509

[19] World Health Organization. International Guide for Monitoring Alcohol Consumption. Geneva: World Health Organization, 2000.

[20] Zou G. A modified poisson regression approach to prospective studies with binary data. Am J Epidemiol. 2004;159(7):702–6. Epub 2004/03/23. . 15033648 10.1093/aje/kwh090

[21] Spiegelman D, Hertzmark E. Easy SAS calculations for risk or prevalence ratios and differences. Am J Epidemiol. 2005;162(3):199–200. Epub 2005/07/01. 10.1093/aje/kwi188 . 15987728

[22] Lambert D. Zero-Inflated Poisson Regression, with an Application to Defects in Manufacturing. Technometrics. 1992;34:1 10.2307/1269547

[23] Min Y, Agrest A. Modeling Nonnegative Data with Clumping at Zero: A Survey. JIRSS. 2002;1:7–33.

[24] Vuong QH. Likelihood Ratio Tests for Model Selection and Non-Nested Hypotheses. Econometrica. 1989;57:307 10.2307/1912557

[25] Bozdogan H. Model selection and Akaike's Information Criterion (AIC): The general theory and its analytical extensions. Psychometrika. 1987;52:345–70. 10.1007/BF02294361

[26] Launer RL, Wilkinson GN. Robustness in Statistics. Launer RL, editor: Elsevier; 1979.

[27] Nijman T, Verbeek M. Nonresponse in panel data: The impact on estimates of a life cycle consumption function. Journal of Applied Econometrics. 1992;7(3):243–57. 10.1002/jae.3950070303

[28] Sirven N, Rapp T. The Dynamics of Hospital Use among Older People Evidence for Europe Using SHARE Data. Health Serv Res. 2017;52(3):1168–84. 10.1111/1475-6773.12518 27319798 PMC5441513

[29] Arrighi Y, Rapp T, Sirven N. The impact of economic conditions on the disablement process: A Markov transition approach using SHARE data. Health Policy. 2017;121(7):778–85. 10.1016/j.healthpol.2017.05.002 . 28527626

[30] Neyeloff JL, Fuchs SC, Moreira LB. Meta-analyses and Forest plots using a microsoft excel spreadsheet: step-by-step guide focusing on descriptive data analysis. BMC Research Notes. 2012;5:52 10.1186/1756-0500-5-52 22264277 PMC3296675

[31] Caliendo M, Lee W-S. Fat chance! Obesity and the transition from unemployment to employment. Economics & Human Biology. 2013;11:121–33. 10.1016/J.EHB.2012.02.002 22391338

[32] Devaux M, Sassi F. The Labour Market Impacts of Obesity, Smoking, Alcohol Use and Related Chronic Diseases. 2015 10.1787/5jrqcn5fpv0v-en.

[33] Han E, Norton EC, Powell LM. Direct and indirect effects of body weight on adult wages. Economics & Human Biology. 2011;9:381–92. 10.1016/J.EHB.2011.07.002 21820369

[34] Devaux M, Sassi F. Alcohol consumption and harmful drinking. 2015 10.1787/5js1qwkz2p9s-en.

[35] Dave D, Kaestner R. Alcohol Taxes and Labor Market Outcomes: National Bureau of Economic Research, Cambridge, MA; 2001. 10.1016/s0167-6296(01)00134-5 12022263

[36] Boden JM, Lee JO, Horwood LJ, Grest CV, McLeod GFH. Modelling possible causality in the associations between unemployment, cannabis use, and alcohol misuse. Social Science & Medicine. 2017;175:127–34. 10.1016/j.socscimed.2017.01.001 . 28088618

[37] Kenkel D, Wang P. Are Alcoholics in Bad Jobs?: National Bureau of Economic Research, Cambridge, MA; 1998.

[38] Jørgensen MB, Thygesen LC, Becker U, Tolstrup JS. Alcohol consumption and risk of unemployment, sickness absence and disability pension in Denmark: a prospective cohort study. Addiction. 2017;112(10):1754–64. 10.1111/add.13875 28544338

[39] Henkel D. Unemployment and substance use: a review of the literature (1990–2010). Curr Drug Abuse Rev. 2011;4(1):4–27. . 21466502 10.2174/1874473711104010004

[40] Morris JK, Cook DG, Shaper AG. Non-employment and changes in smoking, drinking, and body weight. Bmj. 1992;304(6826):536–41. 1559056 10.1136/bmj.304.6826.536 PMC1881409

[41] von Bonsdorff ME, Huuhtanen P, Tuomi K, Seitsamo J. Predictors of employees' early retirement intentions: an 11-year longitudinal study. Occupational Medicine. 2010;60:94–100. 10.1093/occmed/kqp126 19734239

[42] Cortaredona S, Ventelou B. The extra cost of comorbidity: multiple illnesses and the economic burden of non-communicable diseases. BMC medicine. 2017;15:216 10.1186/s12916-017-0978-2 . 29221453 PMC5723100

[43] van Oostrom SH, Gijsen R, Stirbu I, Korevaar JC, Schellevis FG, Picavet HSJ, et al Time Trends in Prevalence of Chronic Diseases and Multimorbidity Not Only due to Aging: Data from General Practices and Health Surveys. PloS one. 2016;11:e0160264 10.1371/journal.pone.0160264 27482903 PMC4970764

[44] Johansson P, Palme M. Assessing the Effect of Public Policy on Worker Absenteeism. The Journal of Human Resources. 2002;37:381 10.2307/3069652

