# Supplementary material for: The short-term effect of BMI, alcohol use, and related chronic conditions on labour market outcomes: A time-lag panel analysis utilizing European SHARE dataset
Source: PLoS One. 2019 Mar 11;14(3):e0211940. doi: 10.1371/journal.pone.0211940 (PMC6411140; doi:10.1371/journal.pone.0211940)
Supplement: S1 File — (DOCX) [file pone.0211940.s001.docx]

## S1: Additional Methods and Results on Measuring the indirect economic burden of disease

The economic burden of risk factors and associated diseases can be estimated through a variety of approaches. A comprehensive review of methodologies confirmed that the majority of studies on the economic burden of disease use the Cost of Illness (COI) approach [1], with origins in the 1960s [2]. Other economic impacts often modelled as part of the economic burden of diseases are the impact on economic output (as measured by GDP and GDP growth), as well as the social or welfare costs of diseases.

In the COI approach, costs are divided into direct and indirect costs, (Fig A).

Figure A: Components of Cost of Illness studies


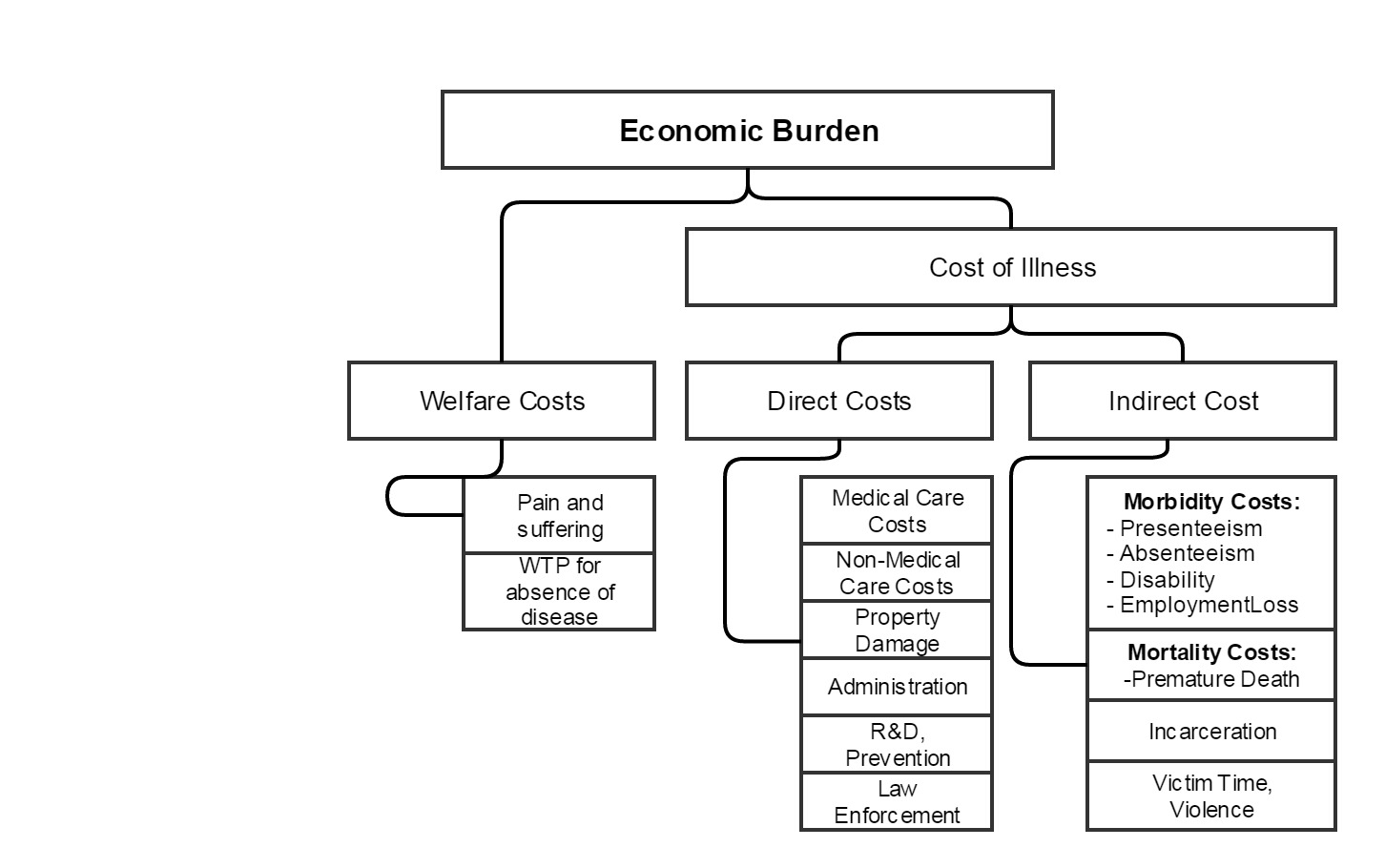


*Note*: This schematic shows that the Cost of Illness can be divided into direct and indirect costs. Direct costs refer to actual payments made directly in relation to the disease in questions. These include the costs of medical care, non-medical care (i.e. active transport to the hospital), property damage in the case of alcohol use, administration costs, the costs for research and development, prevention measures and policy, and the cost of law enforcement, which is particularly relevant in the case of alcohol use. Indirect Costs include the costs arising due to morbidity, such as presenteeism, absenteeism, lost productivity and employment, as well as productivity losses due to premature death. Other indirect costs may include incarceration, violence, and victim time. Victim time refers to mortality costs due to victims of alcohol related crime including time loss for victims of crime, and the cost of victims’ productivity losses.

Direct costs refer to actual payments made directly in relation to the disease in questions, whereas indirect costs are those for which resources are lost without direct payment. Indirect costs may include costs associated with premature mortality, reduced productivity (i.e. absenteeism and presenteeism), employment loss, and victim time (related to alcohol use) (Fig A).

### Range of the indirect burden of alcohol and obesity in high income countries

A structured review revealed ten peer-reviewed and working papers that included the indirect economic impacts of obesity (OECD analyses on France 2016 [3]; Canada 2010 [4]; Australia 2008 [5]; Germany 2011 [6]; Germany 2016 [7]) between 2000 and 2017. Among these ten studies were seven single, and three multi-country studies. Further, two focused on indirect costs, and eight modelled both direct and indirect costs associated with overweight and obesity [7].

There was a large variation for the timeframes for which the results were reported: whereas some studies reported the results on an annual basis [3], other studies reported the future costs of obesity over a given time span [8].

Among the approaches to model the indirect costs associated with obesity, the human capital approach was most common (it was applied by seven studies), a finding that echoes those of other systematic reviews [9, 10]. The impact of indirect costs varied between 0.20 % (Germany 2015) and 1.21% of GDP (Germany 2016). The reason for this wide range is found in the two different methodologies applied to arrive at these results: those pointing to lower indirect costs are based on a prevalence based, population-attributable fraction (PAF) approach, and included cost on sickness absence, early retirement, and early mortality. The results from Germany (2016) were based on a regression approach, and included excess costs due to all healthcare mortality, and sick leave in the German population. The semi-structured review of the evidence on the indirect economic burden of alcohol revealed nine relevant studies between 2000 and 2017. Total indirect costs ranged from 0.19% (Portugal) to 1.6% (Estonia) of GDP in the year the costs were incurred. For most countries for which data were available, the indirect costs of obesity (excluding the social costs) ranged between one and 1.5 percent of GDP.

### Conditions associated with 95% of the overweight/obesity and alcohol related disease burden

| Overweight and Obesity | Data in SHARE? | Alcohol | Data in SHARE? |
| --- | --- | --- | --- |
| Ischemic Heart Disease (IHD) | yes | Cirrhosis | x |
| Diabetes Mellitus (DM) | yes | Alcohol Use disorder | x |
| Cardiovascular Disease (CVD) | yes | Road and unintentional injuries | x |
| Chronic Kidney Disease (CKD) | x | Self-harm | x |
| Low back and neck pain | x | Liver Cancer, Breast Cancer | Yes (any cancer) |
| Hypertensive Heart Disease | yes | CVD | yes |
| Colon and rectum cancer | Any cancer | Nasopharynx & Esophageal Cancer | yes |
| Esophageal cancer |  | *IHD** | *yes* |
| Liver Cancer |  | *DM** | *yes* |
| Kidney and Pancreatic Cancer |  |  |  |

Table A: Conditions associated with 95% of the overweight/obesity and alcohol-related disease burden in the EU-28 +3 – Disease Exposure Variables

*Note:* Note that (moderate) alcohol use is protective of IHD (Ischaemic Heart Disease) and DM (Diabetes Mellitus) based on GBD 2016. Conditions are listed in descending order of contributing to the disease burden.

So*urce:* OECD analysis of Global Burden of Disease 2016 data [11]

### Additional information on methodology

| **BMI Classification [12]** |  |
| --- | --- |
| < 18.5 kg/m^2^ | underweight |
| 18.5 to <25 kg/m^2^ | normal weight |
| 25.0 to <30 kg/m^2^ | overweight |
| >=3030 kg/m^2^ | obese |
| **Binary BMI exposure (salary model)** |  |
| 18.5 to <25 kg/m^2^ | normal weight |
| **Drinking level definition** |  |
| *Categorical classifications* |  |
| Lifetime Abstainer (control) |  |
| Former drinker |  |
| Current drinker | Has consumed alcohol in the past 3 months |
| *Continuous classification among drinkers* |  |
| >20g alcohol/day for women  >40g alcohol/day for men | Heavy drinker, female  Heavy drinker, male |

Table B: Risk Factor Classification

*Note:* The number of standard drinks corresponding to these thresholds varies slightly from country to country, largely because of differences among countries in the ethanol content of a standard.

Indirect costs associated with overweight/obesity and alcohol use often exceed one percent of countries’ annual GDP. Still, few rigorous cross-country studies on indirect costs exist, and study methodologies and data quality vary widely.

| Waves: | 1 | 2 | 4 | 5 | 6 |
| --- | --- | --- | --- | --- | --- |
| Austria | √ | √ | √ | √ | √ |
| Belgium | √ | √ | √ | √ | √ |
| Czech Republic | |  | √ | √ | √ |
| Denmark | √ | √ | √ | √ | √ |
| Estonia |  |  | √ | √ | √ |
| France | √ | √ | √ | √ | √ |
| Germany | √ | √ | √ | √ | √ |
| Greece | √ | √ |  |  | √ |
| Italy | √ | √ | √ | √ | √ |
| Luxembourg | |  |  | √ | √ |
| Netherlands | √ | √ | √ | √ |  |
| Poland |  |  | √ |  |  |
| Portugal |  |  | √ |  | √ |
| Slovenia |  |  | √ | √ | √ |
| Spain | √ | √ | √ | √ | √ |
| Sweden | √ | √ | √ | √ | √ |
| Switzerland | √ | √ | √ | √ | √ |

Table C: List of countries in SHARE 1, 2, 4, 5, and 6

*Note:* SHARE Wave 3 is a historical, retrospective survey, and was not relevant for the purposes of this analysis [13].

### Model Specification

Several modes were tested to study the impact of health status, risk factors, and health behaviours on employment likelihood (See Supplement for additional analytical approaches and robustness checks). The main model to estimate employment likelihood was a Poisson regression (specifying the Incidence Rate Ratio (IRR) options, which approximate Relative Risks), with country level fixed effects, estimating the lagged health outcomes of wave 5 on employment variables in wave 6. Poisson regression has the advantage of approximating relative risks, a more interpretable measure than the odds ratio.

$$ln\left[ Y \right]_{i.t}=\beta_{0}+ {\beta_{1}*x}_{1,t}+\left( \beta_{2}{*\vartheta}_{1, t-1}+\ldots+\beta_{n+2}*\vartheta_{n, t-1} \right)+\beta_{n+3}* {age}_{t}*{sex}_{t}+\left( \beta_{n+4}*x_{2}+\ldots+ {\beta_{n+4+i}x}_{ni} \right)+c+ \varepsilon$$

Where:

$\beta_{0}$ … intercept

$x_{1}$ … time varying covariate

$\vartheta_{1, t-1}$to $\vartheta_{n, t-1}$ … lagged health exposure variables

age*sex … interacted age and sex

$\varepsilon$ … error term

c … country

$x_{2}$ to $x_{n}$ … time invariant covariates (potential confounders, like marital status, education, etc (See S4 Table.)

Y …. Probability of employment in year/wave t

t = time period of conducted SHARE wave

|  | **Outcome** | **Main Exposure** | **Modelling Approach** | **Covariates** | **Lagged Variables** | **LTFU adjustment** | **Sub-analysis** | **Countries in the model** | **Wave** |
| --- | --- | --- | --- | --- | --- | --- | --- | --- | --- |
| 1 | **Employment status** |  | Poisson (IRR option) |  |  |  |  |  |  |
|  |  | Lagged BMI |  | gender, marital status, education, country, ever smoker, physical activity, age | hypertension, diabetes, cancer, lung disease, heart disease, stroke, alcohol use |  |  | AUT, BEL, CZR, DEN, EST, FRA, GER, ITA, NET, POL, POR, ESP, SWE, SUI, LUX, SLO | 5 to 6 |
|  |  | Lagged Alcohol Use |  | gender, age, # social activities per year, smoking status, education, country, physically active, | hypertension, diabetes, cancer, lung disease, heart disease, stroke, BMI | IPW | impact on those employed in previous wave | AUT, BEL, CZR, DEN, EST, FRA, GER, ITA, NET, POL, POR, ESP, SWE, SUI, LUX, SLO | 4 to 5 |
|  |  | Chronic Conditions |  | gender, marital status, education, country, ever smoker, physical activity, age | BMI, alcohol use |  |  | AUT, BEL, CZR, DEN, EST, FRA, GER, ITA, NET, POL, POR, ESP, SWE, SUI | 5 to 6 |
|  |  | at least one/two NCDs |  | gender, marital status, education, country, ever smoker, physical activity, age | BMI, alcohol use |  |  | AUT, BEL, CZR, DEN, EST, FRA, GER, ITA, NET, POL, POR, ESP, SWE, SUI, LUX, SLO | 5 to 6 |
| 2 | **Days of Work missed per year** |  | ZIP |  |  |  |  |  |  |
|  |  | Lagged BMI |  | gender, marital status, education, country, ever smoker, physical activity, age | hypertension, diabetes, cancer, lund disaese, heart disease, stroke, alcohol use |  |  | AUT, BEL, CZR, DEN, EST, FRA, GER, ITA, NET, POL, POR, ESP, SWE, SUI | 1 to 2 |
|  |  | Lagged Alcohol Use |  | gender, age, # social activities per year, smoking status, education, country, physically active, | hypertension, diabetes, cancer, lung disease, heart disease, stroke, BMI | IPW | impact on those employed in previous wave | AUT, BEL, CZR, DEN, EST, FRA, GER, ITA, NET, ESP, SWE, SUI | 4 to 5 |
|  |  | Chronic Conditions |  | gender, marital status, education, country, ever smoker, physical activity, age | BMI, alcohol use |  |  | AUT, BEL, CZR, DEN, EST, FRA, GER, ITA, NET, POL, POR, ESP, SWE, SUI | 1 to 2 |
|  |  | at least one/two NCDs |  | gender, marital status, education, country, ever smoker, physical activity, age | BMI, alcohol use |  |  | AUT, BEL, CZR, DEN, EST, FRA, GER, ITA, NET, POL, POR, ESP, SWE, SUI | 1 to 2 |
| 3 | **Hours of work per week missed** |  | Linear regression |  |  |  |  |  |  |
|  |  | Lagged BMI |  | gender, marital status, education, country, ever smoker | hypertension, diabetes, cancer, lund disaese, heart disease, stroke, alcohol use |  |  | AUT, BEL, CZR, DEN, EST, FRA, GER, ITA, NET, POL, POR, ESP, SWE, SUI, LUX, SLO | 5 to 6 |
|  |  | Lagged Alcohol Use |  | gender, age, # social activities per year, smoking status, education, country, physically active, | hypertension, diabetes, cancer, lung disease, heart disease, stroke, BMI | IPW |  | AUT, BEL, CZR, DEN, EST, FRA, GER, ITA, NET, POL, POR, ESP, SWE, SUI, LUX, SLO | 4 to 5 |
|  |  | Chronic Conditions |  | gender, marital status, education, country, ever smoker, past alcohol use | BMI, alcohol use |  |  | AUT, BEL, CZR, DEN, EST, FRA, GER, ITA, NET, POL, POR, ESP, SWE, SUI, LUX, SLO | 5 to 6 |
|  |  | at least one/two NCDs |  | gender, marital status, education, country, ever smoker, past alcohol use | BMI, alcohol use |  |  | AUT, BEL, CZR, DEN, EST, FRA, GER, ITA, NET, POL, POR, ESP, SWE, SUI, LUX, SLO | 5 to 6 |
| 4 | **Intention to retire early** |  | Poisson (IRR option) |  |  |  |  |  |  |
|  |  | Lagged BMI |  | gender, marital status, education, country, ever smoker, physical activity, home ownership, age | hypertension, diabetes, cancer, lund disaese, heart disease, stroke, alcohol use |  |  | AUT, BEL, CZR, DEN, EST, FRA, GER, ITA, NET, POL, POR, ESP, SWE, SUI, LUX, SLO | 5 to 6 |
|  |  | Lagged Alcohol Use |  | gender, age, # social activities per year, smoking status, education, country, physically active, | hypertension, diabetes, cancer, lung disease, heart disease, stroke, BMI | IPW |  | AUT, BEL, CZR, DEN, EST, FRA, GER, ITA, NET, ESP, SWE, SUI | 4 to 5 |
|  |  | Chronic Conditions |  | gender, marital status, education, country, ever smoker, physical activity, home ownership, age | BMI, alcohol use |  |  | AUT, BEL, CZR, DEN, EST, FRA, GER, ITA, NET, POL, POR, ESP, SWE, SUI, LUX, SLO | 5 to 6 |
|  |  | at least one/two NCDs |  | gender, marital status, education, country, ever smoker, physical activity, home ownership, age | BMI, alcohol use |  |  | AUT, BEL, CZR, DEN, EST, FRA, GER, ITA, NET, POL, POR, ESP, SWE, SUI, LUX, SLO | 5 to 6 |

Table D: Model Specifications

### Inverse Probability Weights for the Alcohol Module

Alcohol consumption variables in SHARE are consistent for Waves 4 and 5, but had been updated in Wave 6. Therefore, Waves 4 and 5 were chosen for the analysis of the impact of alcohol use on the studied labour market outcomes.

However, since there was ~25% attrition of working-age individuals between wave 4 and 5, it was tested whether attrition was associated with employment status, and among those who worked in wave 4, associated with absenteeism and intention to retire early. All outcome variables were significantly associated with attrition, therefore missingness (attrition) at random with respect to the outcomes variables was ruled out, highlighting the need to adjust for loss-to-follow-up bias.

To address this, inverse probability weights were created to adjust for missingness among the work-eligible and working population in Wave 5, versus Wave 4. The methods of stabilized IPW was chosen, and the denominator of the weight as estimated by estimating the likelihood of loss to follow up based on age, gender, alcohol use (Former/abstainer/moderate/binge), ever smoker, marital status, working status (for weights for employment likelihood estimation), educational attainment, COPD, stroke, heart disease, hypertension, diabetes, cancer, with clustering at the country level.

The numerator of the weight was the probability of missingness. The specifics of the stabilized weight are presented in the table below (Table E).

Creating of IPW (Inverse Probability Weights)

| Stabilized IPW for Wave 4-5 Alcohol Module |  |  |  |
| --- | --- | --- | --- |
|  | **Weight** | **Range** | **SD** |
| Weight for missingness for working-eligible population | .9924 | 0.8176 to 1.345 | .0719 |

Table E: Details on Inverse Probability Weights

### Country-specific results of employment likelihood

The first explored model to study the impact of lagged health status on employment likelihood was a pooled logistic regression, where the impact of lagged health outcomes from waves 1 and 5 on employment outcomes in waves 2 and 6 were assessed. A variable specifying the ‘era’ from which the data originated was included in the model.

Tests for effect modification for each exposure and control variable were performed, as well as for the impact of ‘era’. Since the effects (except for hypertension and stroke) of a previous health status on employment likelihood was not affected by era, but since significant pension and retirement reforms had occurred between the first and second and the fifth and sixth wave, only the impact of the fifth on the sixth wave was modelled for the main results.

Fixed effects logistics regressions were alternative models for model number one, and lagged Poisson regressions alternate models for model two. Further, dynamic fixed effect models were explored for waves that did not require adjustment for missingness patterns. However, convergence remained an issue for dynamic fixed effects models.

Figure B. Employment likelihood due to BMI


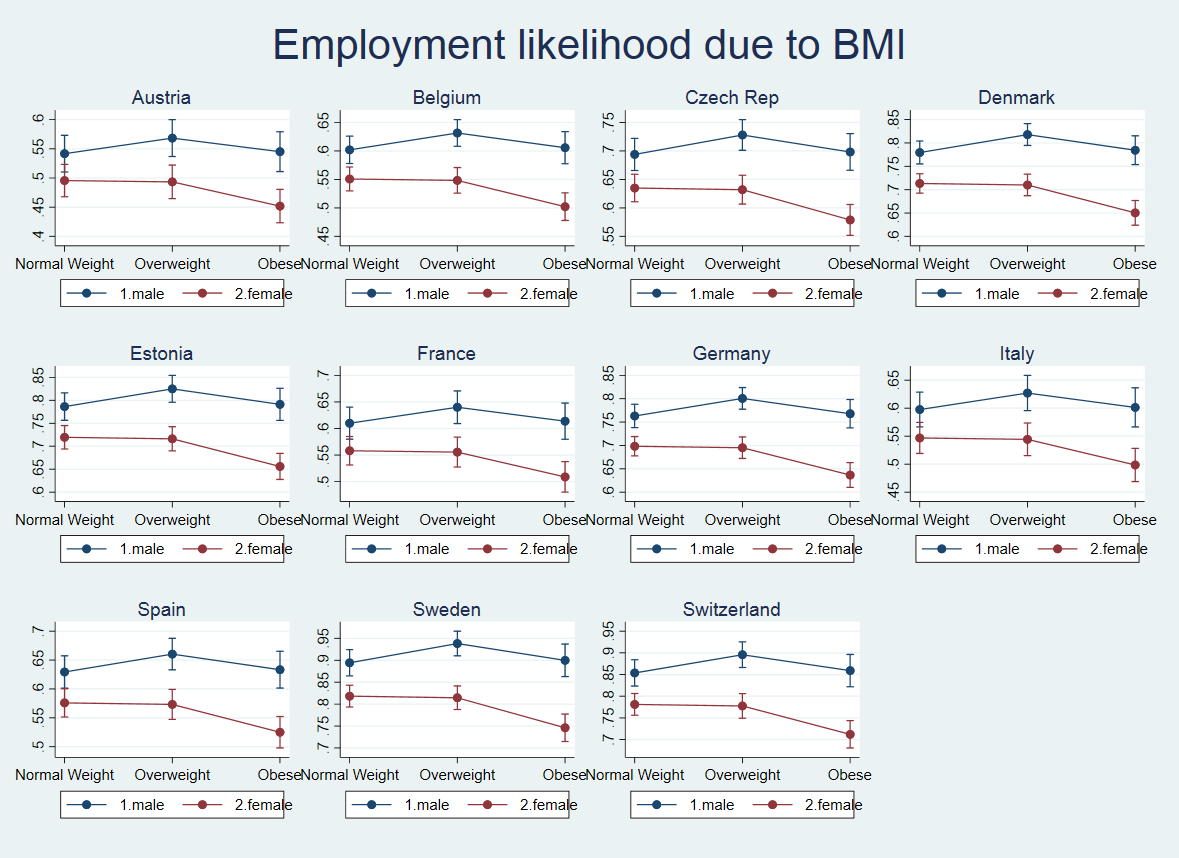


*Note*: This figure shows the probabilities of being employed in men and women under three different BMI categories (1=normal weight; 2 = overweight; 3 = obese). Although country specific employment probabilities differ, employment probabilities are highest in overweight men, and lowest in obese women. Overall employment probabilities are consistently lower among women, in all modelled countries. . The model was adjusted for marital status, age, education, country level fixed effects, level of physical activity, smoking, drinking, and other chronic diseases.

.

Figure C. Employment likelihood due to Lung Disease/COPD


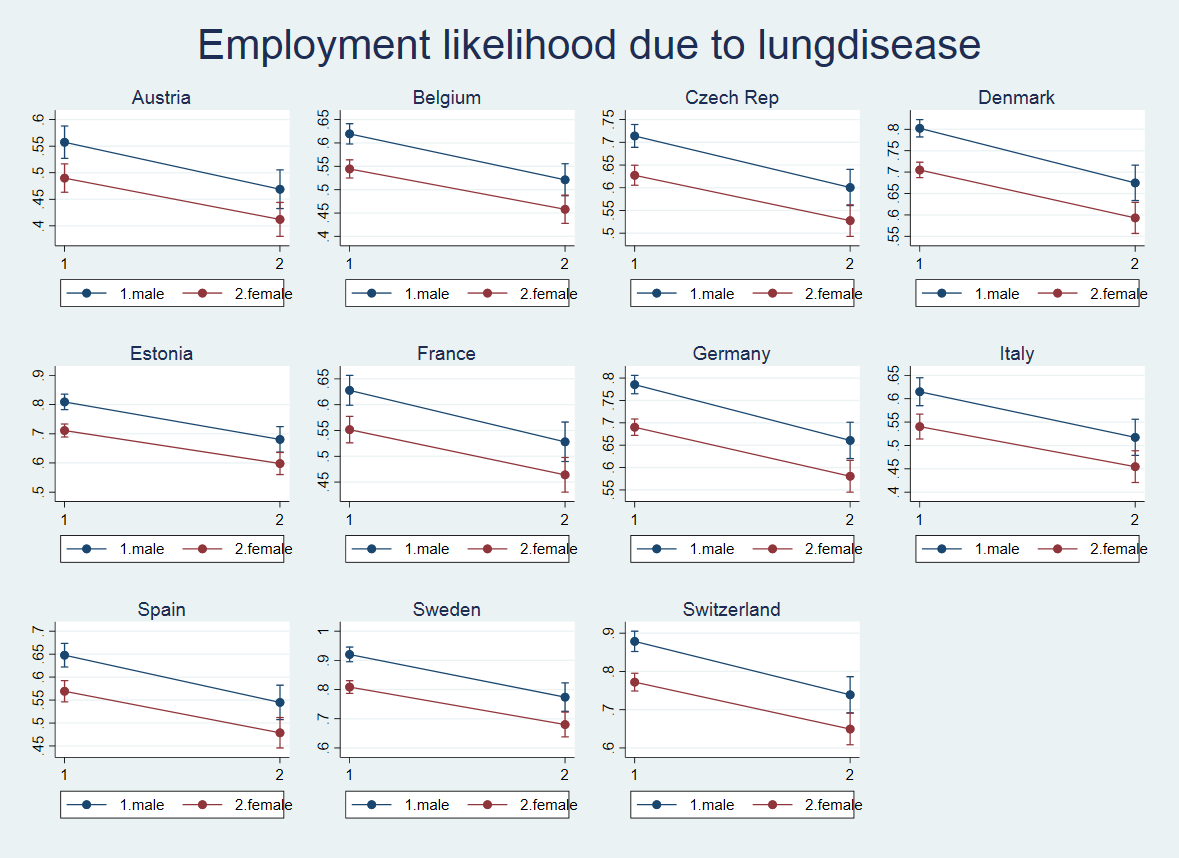


*Note*: This figure shows the probabilities of being employed in men and women with and without COPD and lung disease. Although country specific employment probabilities differ, employment probabilities are highest in men free of lung disease, and lowest in women with COPD. Overall employment probabilities are consistently lower among women, in all modelled countries.

Figure D. Employment Likelihood due to hypertension


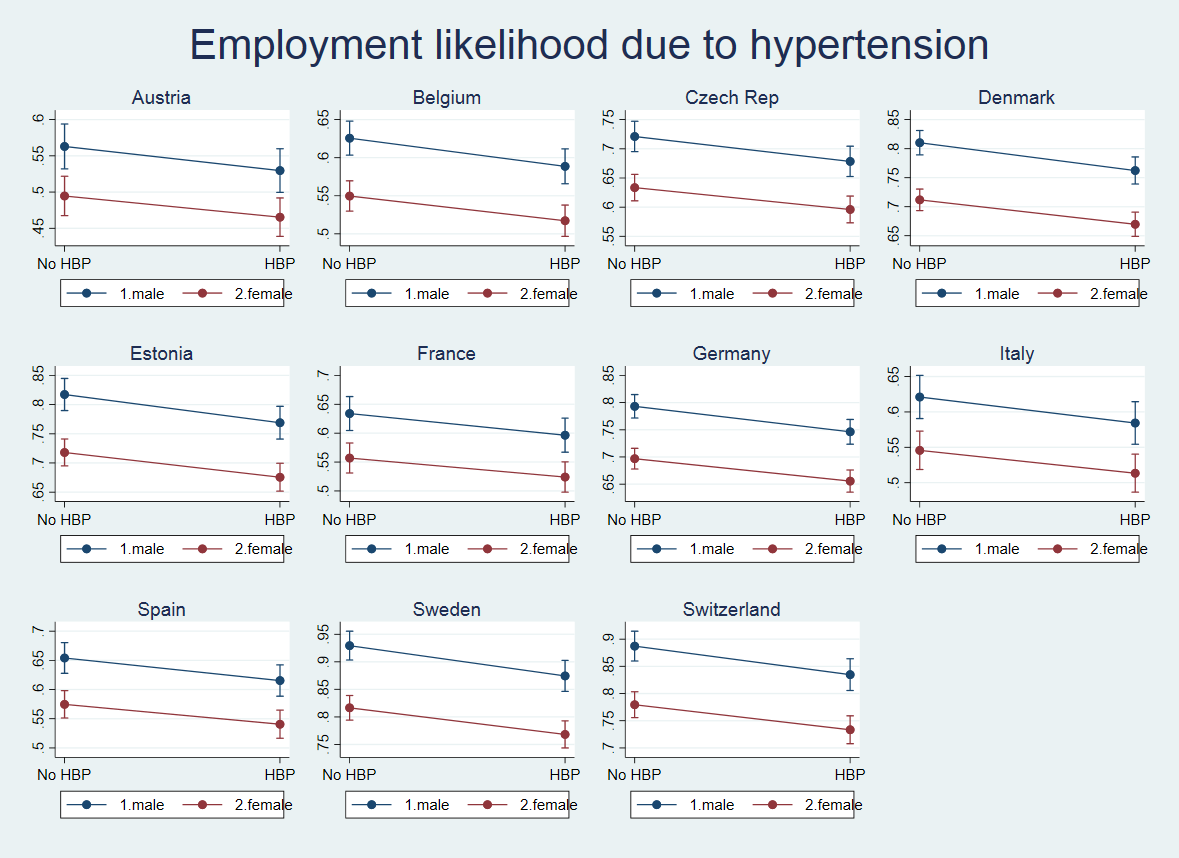


*Note*: This figure shows the probabilities of being employed in men and women with and without hypertension. Although country specific employment probabilities differ, employment probabilities are highest in men with no hypertension, and lowest in hypertensive women. Overall employment probabilities are consistently lower among women, in all modelled countries. Note that high blood pressure is defined as a systolic/diastolic blood pressure of ≥140/90 mm Hg.

Figure E. Probability of being employed given previous cancer diagnosis


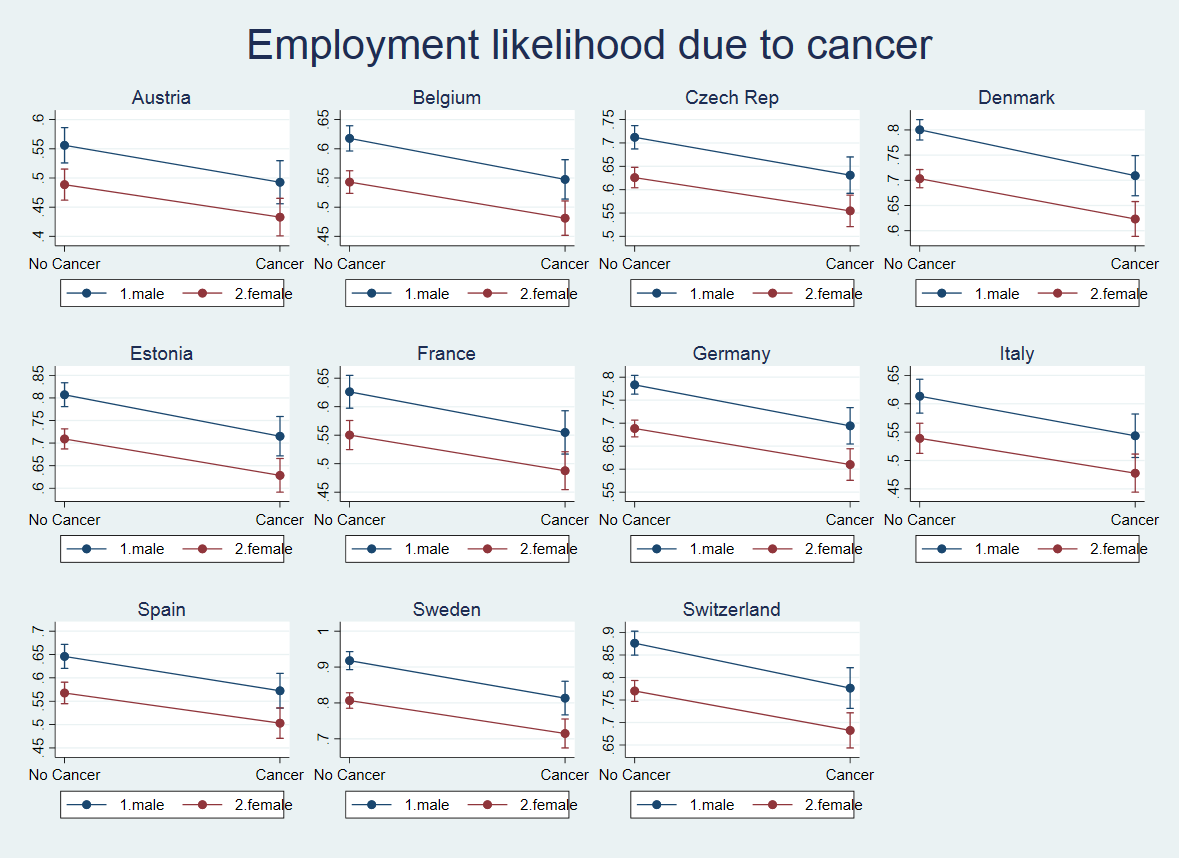


*Note*: This figure shows the probabilities of being employed in men and women and without cancer. Although country specific employment probabilities differ, employment probabilities are highest in men without cancer, and lowest among women previously diagnosed with cancer. Overall employment probabilities are consistently lower among women, in all modelled countries. All models are adjusted for age, levels of physical activity, diseases in previous year, education, marital status, and smoking status.

Analysis of Harmonized SHARE + SHARE employment module, Release 6.0.

Women with a previous cancer diagnosis have lowest employment probability compared to both men with and without such diagnosis, a result that is significant compared to men suffering from cancer in Denmark, Estonia, Germany, Italy, Sweden, and Switzerland (Fig E).

Similar results are seen for employment probabilities after a heart disease diagnosis (Fig F). Employment probabilities in men and women without heart disease were statistically significantly different in all modelled countries, whereas this statistical significance in the presence of heart disease persisted in Germany, Sweden, Switzerland, and Denmark, but not in the other modelled countries. Thus, there seems to be a convergence in employment likelihood across gender in the presence of heart disease.

Figure F. Employment probability in women and men with heart disease


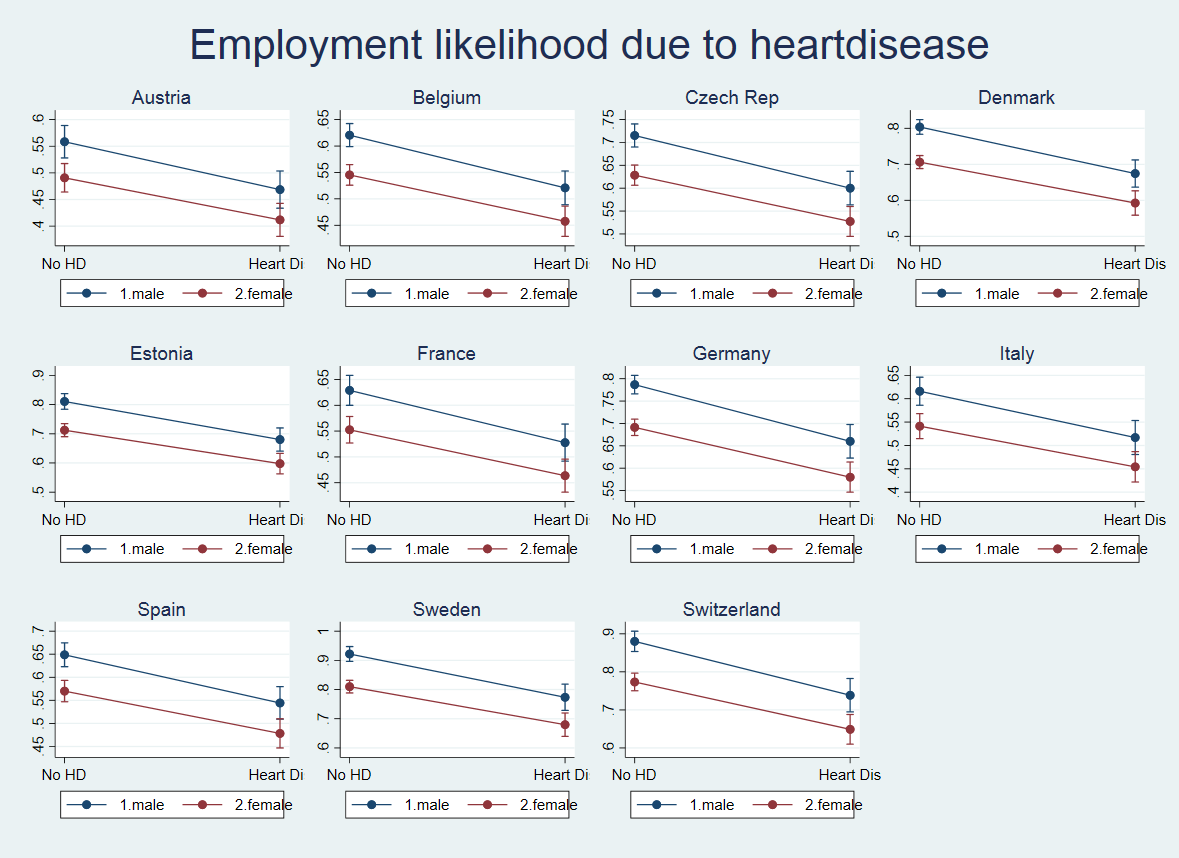


*Note*: This figure shows the probabilities of being employed in men and women with and without heart disease. Although country specific employment probabilities differ, employment probabilities are highest in men without the condition, and lowest in women with heart disease. Overall employment probabilities are consistently lower among women, in all modelled countries. All models are adjusted for age, levels of physical activity, diseases in previous year, education, marital status, and smoking status.

Figure G. Employment likelihood due to diabetes


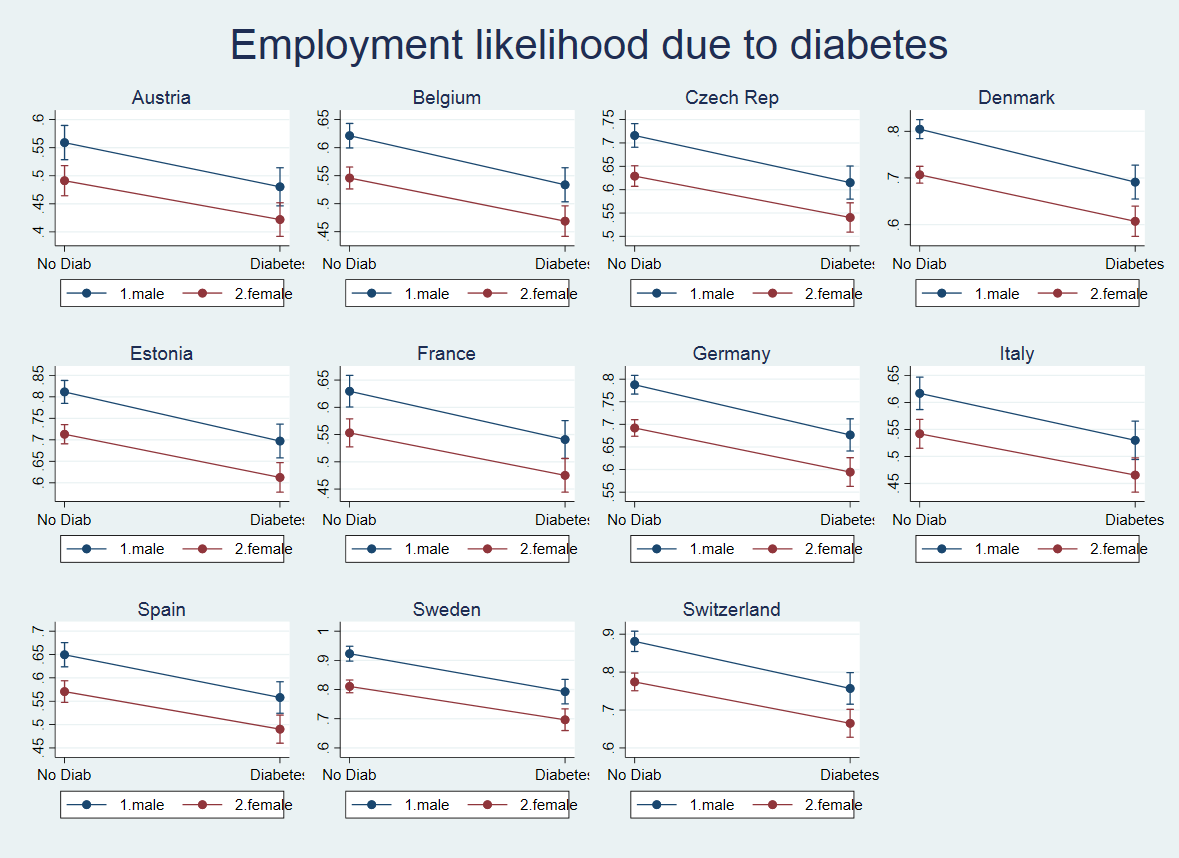


*Note*: This figure shows the probabilities of being employed in men and women with and without diabetes. Although country specific employment probabilities differ, employment probabilities are highest men without diabetes (a significant result for all modelled countries), and lowest in female diabetes. Overall employment probabilities are consistently lower among women, in all modelled countries. All models are adjusted for age, levels of physical activity, diseases in previous year, education, marital status, and smoking status.

### Additional Results on Early Retirement Wish due to high BMI by Gender

Figure H. Obese women express higher interest in early retirement

*Note*: This Figure reflects the impact of obesity/overweight by gender in the analysis of the early retirement wish given a higher than normal BMI. Analysis based on a Poisson model, adjustment for marital status, country, education, smoking, alcohol consumption, presence of chronic diseases, and level of physical activity. Analysis conducted on Wave 5 and 6 of SHARE, on subsample of those who were in the active working population in Wave 5, and expected to participate in the working population in Wave 6.

### Additional Results on the impact of NCDs on Absenteeism at the Country Level

Figure I. Days of work missed due to Diabetes

*Note*: Results based on zero-inflated Poisson model. The average days of work missed was 9.3. Workers in Spain missed most days due to diabetes (15.4), versus workers in Italy missed the lowest amount. The model was adjusted for marital status, age, education, country level fixed effects, other confounders. The individual country results were statistically significant at p<0.05 for all countries.

Fig I represents the marginal effects of diabetes on absenteeism – that is, the additional days that are missed when a person had diabetes in the previous period, compared to those who did not. Fig J represents the additional days missed per year due to cancer. For an illustration of total days of work missed with and without cancer, please see Fig K.

Figure J. Days of work missed due to cancer

*Note*: Results based on zero-inflated Poisson model. The average days of work missed was 8.7. Workers in Spain missed most days due to cancer (14.2), versus workers in Italy missed the lowest amount. This Figure represents the marginal effects of cancer on absenteeism – that is, the additional days that are missed when a person had cancer in the previous period, compared to those who did not. The model was adjusted for marital status, age, education, country level fixed effects, other confounders. The results shown in this graph are statistically significant at p<0.05 overall, and at p<0.1 at the country-specific level; the results for Sweden are statistically significant at p<0.05.

Figure K. Total missed days at work due to previous cancer diagnosis

### References

1. World Health Organization. WHO guide to identifying the economic consequences of disease and injury. Geneva: 2009.

2. Rice DP. Estimating the cost of illness. American Journal of Public Health and the Nations Health. 1967;57:424-40.

3. Ministère de l’Économie et des Finances. Obésité : quelles conséquences pour l'économie et comment les limiter? Paris: Ministère de l’Économie et des Finances, 2016.

4. Anis AH, Zhang W, Bansback N, Guh DP, Amarsi Z, Birmingham CL. Obesity and overweight in Canada: an updated cost-of-illness study. Obes Rev. 2010;11:31-40. doi: 10.1111/j.1467-789X.2009.00579.x. PubMed PMID: 19413707.

5. Access Economics. The growing cost of obesity in 2008: three years on. Access Economics Pty Limited, 2008.

6. Konnopka A, Bodemann M, Konig HH. Health burden and costs of obesity and overweight in Germany. Eur J Health Econ. 2011;12:345-52. doi: 10.1007/s10198-010-0242-6. PubMed PMID: 20401679.

7. Effertz T, Engel S, Verheyen F, Linder R. The costs and consequences of obesity in Germany: a new approach from a prevalence and life-cycle perspective. Eur J Health Econ. 2016;17:1141-58. doi: 10.1007/s10198-015-0751-4. PubMed PMID: 26701837.

8. Lightwood J, Bibbins-Domingo K, Coxson P, Wang YC, Williams L, Goldman L. Forecasting the future economic burden of current adolescent overweight: an estimate of the coronary heart disease policy model. Am J Public Health. 2009;99:2230-7. doi: 10.2105/AJPH.2008.152595. PubMed PMID: 19833999.

9. Thavorncharoensap M, Teerawattananon Y, Yothasamut J, Lertpitakpong C, Thitiboonsuwan K, Neramitpitagkul P, et al. The economic costs of alcohol consumption in Thailand, 2006. BMC Public Health. 2010;10:323. doi: 10.1186/1471-2458-10-323. PubMed PMID: 20534112.

10. Rehm J, Baliunas D, Borges GLG, Graham K, Irving H, Kehoe T, et al. The relation between different dimensions of alcohol consumption and burden of disease: an overview. Addiction. 2010;105:817-43.

11. Disease GBD, Injury I, Prevalence C. Global, regional, and national incidence, prevalence, and years lived with disability for 328 diseases and injuries for 195 countries, 1990-2016: a systematic analysis for the Global Burden of Disease Study 2016. Lancet. 2017;390(10100):1211-59. doi: 10.1016/S0140-6736(17)32154-2. PubMed PMID: 28919117; PubMed Central PMCID: PMCPMC5605509.

12. Organization WH. WHO BMI Classification.

13. SHARE. SHARE waves and years 2018 [cited 2018]. Available from: <http://www.share-project.org/data-documentation/waves-overview.html>
